# Ethyl Acetate Extracts of Edible Mushroom Species Enhance β-Lactam Activity Against an MRSA Isolate *in vitro*

**DOI:** 10.17113/ftb.64.02.26.9283

**Published:** 2026-06-15

**Authors:** James Blee, Peter O’Hara, Thomas Smyth, Owen Kenny

**Affiliations:** Department of Health and Nutritional Science, Atlantic Technological University, Sligo, Ireland

**Keywords:** antibiotic modulation, edible mushrooms, MRSA, β-lactams, *Agaricus bisporus*, antimicrobial resistance

## Abstract

**Research background:**

The rapid emergence of antimicrobial resistance (AMR) has led to numerous pathogens developing resistance to available antibiotics. *Staphylococcus aureus* is a leading cause of hospital-acquired infections, and the emergence of resistant strains like methicillin-resistant *Staphylococcus aureus* (MRSA) has led to increased clinical complications and higher mortality rates. As such, restoration of first-line β-lactam antibiotic activity is a viable strategy to combat AMR. The antimicrobial activity of edible mushroom extracts is well reported; however, their use as a source of antibiotic modulators is relatively unexplored, and current reports often do not adhere to drug combination guidelines. The aim of this study is to evaluate the potential of extracts derived from ten edible mushroom species to modulate β-lactam antibiotics against MRSA with reference to the recommended guidelines.

**Experimental approach:**

Antimicrobial activity (minimum inhibitory concentration (MIC)) and antibiotic-modulating activity (fractional inhibitory concentration index (FICI)) were assessed using the broth microdilution method. The cell viability assay was used to assess cell membrane integrity following treatment to offer insight into potential mechanisms of action.

**Results and conclusions:**

Both antimicrobial and antibiotic modulatory activity were found only in ethyl acetate extracts. *Hericium erinaceus* extract showed the strongest antimicrobial activity against *S. aureus* (MIC=0.8 mg/mL), while *Agaricus bisporus* extract exhibited the strongest antibiotic-modulating properties, exhibiting a 25-fold reduction in the MIC of ampicillin, amoxicillin and penicillin against an MRSA isolate (FICI=0.29), indicating synergism. The mechanism of action indicates reduced cell membrane integrity, which may increase intracellular antibiotic concentration.

**Novelty and scientific contribution:**

These results indicate the potential use of ethyl acetate extracts from edible mushrooms, specifically *A. bisporus,* as a source of antibiotic-modulating compounds. To our knowledge, this is the first study to evaluate the antibiotic-modulating effects of *H. erinaceus*, *Hypsizygus tessulatus and Pholiota adiposa.* This study also highlights the necessity to adhere to FICI guidelines when evaluating the antibiotic-modulating potential of edible mushroom extracts.

## INTRODUCTION

The rapid emergence of antimicrobial resistance (AMR) has led to numerous pathogens developing resistance to one or more available antibiotics (*1*). AMR is further complicated by a limited antibiotic pipeline, suggesting that extending the lifespan of currently available antibiotics is a necessary approach. *Staphylococcus aureus* is the leading cause of numerous hospital-acquired (HA) and community-acquired (CA) infections, including impetigo, endocarditis and bloodstream infections (*2*). The annual incidence rate of *S. aureus* bloodstream infections is estimated at approx. 26.1 per 100 000 persons (*3*), with a typical mortality rate of 10–30 % (*4*). Resistance to penicillin has been reported in over 90 % of *S. aureus* isolates (*5*), while methicillin-resistant *Staphylococcus aureus* (MRSA) is estimated to account for 13–74 % of all *S. aureus* infections (*6*). The emergence of MRSA strains and the need for new antibiotic development have been noted by the World Health Organisation, classifying MRSA as a ‘high priority pathogen’ due to its exhibited resistance to numerous antibiotics (*7*). Typically, β-lactam antibiotics are the primary treatment options for methicillin-sensitive *Staphylococcus aureus* (MSSA) infections, which demonstrate no resistance. β-Lactam resistance complicates drug administration, which is indicative of the higher mortality rates associated with MRSA bacteraemia infections than with MSSA infections (*8*). The widespread resistance to β-lactam antibiotics, coupled with a limited antibiotic pipeline, requires an alternative therapeutic approach.

One such therapeutic option is the reutilisation of the β-lactam antibiotics *via* their use with antibiotic modulators. Antibiotic modulators are compounds capable of overcoming resistance mechanisms expressed by pathogens, consequently increasing antibiotic efficacy by lowering its minimum inhibitory concentration (MIC), thus having the potential to extend the lifespan of an antibiotic (*9*). *S. aureus* expresses two predominant resistance mechanisms to β-lactam antibiotics: enzymatic hydrolysis and alteration of the β-lactam substrate, penicillin-binding protein 2 (PBP2). In resistant isolates, the *mecA* gene encodes for PBP2a, which exhibits reduced affinity to β-lactam antibiotics (*10*). Clavulanic acid is a notable example of a natural product-derived antibiotic modulator, capable of serving as a competitive and irreversible inhibitor of serine β-lactamases, rendering the serine β-lactamase ineffective (*11*). The combination of clavulanic acid as a modulator with amoxicillin gave rise to Augmentin, which expanded potential treatment options due to overcoming resistance mechanisms.

Initially derived from fungi, the β-lactams are one of the most important antibiotic classes in modern medicine. As such, there has been increased research interest in exploring edible mushrooms as a source of potential antimicrobial agents. A variety of edible mushroom species, such as the *Pleurotus* spp., have previously demonstrated activity against both Gram-positive (*Bacillus subtilis* and *Streptococcus faecalis*) and Gram-negative (*Escherichia coli* and *Pseudomonas aeruginosa*) bacteria by zone-of-inhibition (disc diffusion) (*12*). However, to date, there are minimal studies reporting on the antibiotic-modulating properties of edible mushroom extracts. Further, the methods used to conduct and classify synergism within the few studies conducted often do not adhere to the recommended classifications (*13*), prompting incomparable results. For example, synergism was previously reported following a 4-fold reduction in meropenem minimum inhibitory concentration (MIC) using *φ*(ethanol)=60 % extracts derived from *Pleurotus ostreatus* against *E. coli* and *Klebsiella pneumoniae* (*14*). However, subinhibitory concentrations of extracts were not used, while the parameters and calculation for defining synergism based on the fractional inhibitory concentration index (FICI≤1) are different from the recommended classification (*13*).

Despite evidence supporting the antimicrobial properties of edible mushroom extracts, the reported MICs are often too high to be considered as independent antimicrobial agents. To date, there are minimal studies reporting on the β-lactam-modulating potential of edible mushroom extracts against MRSA. Effective β-lactam modulation against MRSA may aid in inhibiting or counteracting MRSA resistance mechanisms while extending the lifespan of current β-lactam antibiotics.

Therefore, this study aims to evaluate the antibiotic-modulating potential of extracts derived from ten edible mushroom species against a clinical MRSA isolate.

## MATERIALS AND METHODS

### Chemicals and reagents

Organic solvents (hexane, methanol, dimethyl sulfoxide (DMSO), acetone, and ethyl acetate) were purchased from Lennox laboratory supplies (Dublin, Ireland). The β-lactam antibiotics (penicillin, oxacillin, ampicillin, and amoxicillin), nitrocefin discs, Mueller-Hinton broth and agar, propidium iodide (PI), 4′,6-diamidino-2-phenylindole (DAPI) and iodonitrotetrazolium chloride (INT) were purchased from Sigma-Aldrich, Merck (Dublin, Ireland).

### Sample preparation

Commercially available mushroom species were sourced and purchased from Garryhinch Wood Exotic Mushrooms, Co. Offaly, Ireland in November 2021. A fresh mass of 1 kg of mushrooms *Agaricus bisporus* (portobello), *Grifola frondosa* (maitake), *Hericium erinaceus* (lions mane), *Hypsizygus tessulatus* (white beech), *Lentinula edodes* (shiitake), *Pholiota adiposa* (chestnut), *Pholiota microspora* (forest nameko), *Pleurotus citrinopileatus* (golden oyster), *Pleurotus eryngii* (king oyster) and *Pleurotus ostreatus* (oyster) was freeze dried (model FD 80; Cuddon Freeze Dry, Blenheim, New Zealand) at Teagasc Ashtown Food Research Centre, Dublin, Ireland and then blended into a fine powder (variable speed laboratory blender; Waring, Torrington, CT, USA). Samples were vacuum packed and stored at −20 °C until further use.

### Sample extraction

Each freeze-dried mushroom powder (20 g) was exhaustively extracted (1:10) using a sequence of solvents of increasing polarity (hexane<methanol<water). Samples were extracted with each solvent, under agitation (150 rpm) and at room temperature, for two consecutive two-hour periods, followed by one sixteen-hour extraction. Extracts were pooled according to their respective solvent and subsequently dried using a rotary evaporator (Rotavapor R-3000; Buchi, Flawil, Switzerland) at 40 °C, while water extracts were freeze dried (Alpha 1-2 LD plus; Martin Christ GmbH, Osterode am Harz, Germany) and transferred to amber vials. Dried methanol extracts were reconstituted in water and then subjected to three successive rounds of liquid-liquid partitioning using 250 mL of ethyl acetate in each case. The organic solvent and water were removed as previously described. All extracts were stored at −20 °C until further use.

### Bacterial strains and culture conditions

*Staphylococcus aureus* (ATCC 25923) was purchased from the American Type Culture Collection (ATCC). A hospital-acquired (HA) MRSA isolate was donated by Sligo University Hospital (SUH), Sligo, Ireland. All strains were stored on ceramic beads in glycerol at −80 °C. Prior to use, beads were streaked onto fresh nutrient agar plates and grown for 24 h at 37 °C. Three colonies of similar morphology were inoculated into 15 mL of Mueller-Hinton broth and incubated for 16 h at 37 °C. The inoculum was adjusted to a final testing value of 5·10^5^ CFU/mL, prior to testing. The MRSA isolate was positively typed for the *mecA* gene by SUH. MRSA tested positive, while *S. aureus* tested negative for β-lactamase production, following nitrocefin application according to the manufacturer’s guidelines (Merck, Dublin, Ireland).

### Antimicrobial activity

The broth microdilution method was used to determine the antimicrobial activity of extracts, as described previously by Smyth *et al.* (*15*). Stock solutions (8 mg/mL) of each sample extract were prepared by initially solubilising in sterile deionised water with *φ*(DMSO)=8 %. Extracts were sonicated (Elmasonic S180 sonicator (37 kHz); Elma Ultrasonic, Singen, Germany) for 10 min and centrifuged at 2600×*g* for 5 min using a Sorvall ST4R plus centrifuge (Thermo Fisher Scientific, Waltham, MA, USA).

Using a 96-well microtiter plate, the following controls were added in triplicate to lane one: 100 µL of Mueller-Hinton Broth (blank control), 100 µL of ampicillin (0.5 mg/mL, positive control), 100 µL of bacterial culture and a 4 % (*V*/*V*) aqueous DMSO solution (final concentration for lane 1, negative control). In the remaining wells, a 2-fold serial dilution of each extract was performed in triplicate across corresponding rows using Mueller-Hinton broth, resulting in a final extract concentration of 4 mg/mL. A volume of 100 µL of bacterial culture (5·10^5^ CFU/mL) was added to all corresponding wells, excluding the blank control. Plates were tested in triplicate and were incubated at 37 °C for 18 h. Following incubation, 40 µL of iodonitrotetrazolium dye (*γ*(INT)=0.2 mg/mL) was added to each well to assess cell viability, where viable bacterial cells are responsible for the witnessed colour change from clear to red. The wells where no colour change was observed after incubation were determined as the MIC using biological replicates (*N*=3).

### Antibiotic modulation

Extracts were evaluated for their ability to modulate four β-lactam antibiotics (ampicillin, amoxicillin, oxacillin and penicillin) against an MRSA isolate using the fractional inhibitory concentration index (FICI), as described previously by Embaby *et al*. (*16*). Extracts were diluted to one quarter their respective MIC and added to subtherapeutic concentrations of antibiotic in triplicate, where they were then incubated with an inoculum of 5·10^5^ CFU/mL at 37 °C for 18 h. Equivalent extract volume fractions of DMSO (2 % final) were added to the negative controls containing subtherapeutic concentrations of antibiotic. Cell viability was assessed as previously described using biological replicates (*N*=3). Antibiotic modulation was classified as synergistic (<0.5), additive (0.5–1), indifferent (>1–4), and antagonistic (>4) according to the FICI (*13*):


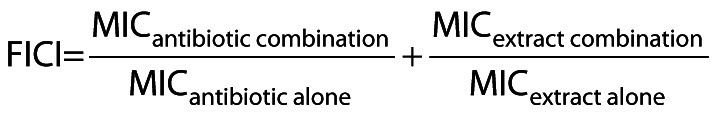
 /1/

### Cell viability assay

The cell viability assay was used to determine cell membrane leakage, as previously described by Khamrai *et al*. (*17*) with minor modifications. A single MRSA colony was inoculated in Mueller-Hinton broth and incubated at 37 °C until mid-logarithmic stage absorbance *A*_600 nm_=0.5 was reached. Next, 800 μL of culture were added to all Eppendorf tubes. The remaining 200 μL was comprised of the antibiotic/extract concentration, while volume fraction of 1 % DMSO was added to the negative control to serve as the vehicle control. Samples were incubated for 1 h at 37 °C under agitation at 150 rpm. Following incubation, samples were centrifuged at 8000×*g* for 8 min (Prism microcentrifuge; Labnet International, Edison, NJ, USA), where the supernatant was discarded and the pellet resuspended in 800 μL of Ringers solution. Then, 100 μL of DAPI solution (0.1 mg/mL) and 100 μL of PI (0.1 mg/mL) were added and incubated in the dark for 30 min at 37 °C under agitation (150 rpm). Following incubation, 10 μL of sample were analysed using a Canon EOS 1300D (Tokyo, Japan) camera attached to Nikon Eclipse 80i (Tokyo, Japan) for fluorescent microscopy. EOS utility software (v. 3.7.0.0) was used for capturing images (400× magnification) (ISO 100) with ND4 filter applied to remove background fluorescence. Daptomycin was used as a positive control. All samples and controls were tested as biological triplicates (*N*=3).

### Total phenolic content

The total phenolic content of all crude extracts was evaluated (*N*=3) as previously described by Kenny *et al*. (*18*). Briefly, 100 μL of extract solubilised in methanol were added to a microcentrifuge tube, followed by 100 μL of methanol, 100 μL of Folin-Ciocalteu (2 N) reagent and 700 μL of sodium carbonate (20 % *m/V*). A volume of 100 μL of methanol was used in place of the extract for the blank control. Each microcentrifuge tube was vortexed and incubated in the dark, at room temperature, for 20 min. Samples were then centrifuged at 16 000×*g* (Prism microcentrifuge; Labnet International) for 3 min and the absorbance was measured at 735 nm using a spectrophotometer (model 6300; Jenway, Essex, UK). A gallic acid calibration curve (0.01–0.2 mg/mL) was used to express the results as gallic acid equivalents (GAE) on dry mass extract basis (mg/mg) following linear regression.

## RESULTS AND DISCUSSION

From the forty extracts screened for their antimicrobial activity, only the ethyl acetate extracts from seven species (*Agaricus bisporus, Hericium erinaceus, Hypsizygus tessulatus, Pholiota adiposa, Pleurotus citrinopileatus, Pleurotus eryngii* and *Pholiota microspora*) demonstrated antimicrobial activity (MIC=0.8–4 mg/mL) against both *S. aureus* and an MRSA isolate (Table 1). No antimicrobial activity was observed in any extracts from *Grifola frondosa*, *Lentinula edodes* or *Pleurotus ostreatus*. The strongest antimicrobial activity was present in *H. erinaceus* ethyl acetate extract against *S. aureus* (0.8 mg/mL). Overall, the tested crude edible mushroom extracts exhibited weak antimicrobial activity. However, extracts demonstrating weak antimicrobial activity (4 mg/mL) displayed the strongest antibiotic modulation against an MRSA isolate, suggesting their potential alternative use.

**Table 1:** The antimicrobial activity (MIC) of ethyl acetate extracts derived from edible mushrooms against two bacterial isolates

Species	*S. aureus* ATCC 25923	HA-MRSA
	MIC/(mg/mL)	
*Agaricus bisporus*	4	4
*Grifola frondosa*	>4	>4
*Hericium erinaceus*	0.8	4
*Hypsizygus tessulatus*	4	4
*Lentinula edodes*	>4	>4
*Pholiota adiposa*	4	4
*Pleurotus citrinopileatus*	1	1
*Pleurotus eryngii*	4	4
*Pholiota microspora*	1	1
*Pleurotus ostreatus*	>4	>4

>4 indicates no activity at the maximum concentration tested (4 mg/mL). Values are means of replicate assays (*N*=3). HA-MRSA=hospital acquired methicillin-resistant *Staphylococcus aureus,* MIC=minimum inhibitory concentration

Of all tested extracts, five ethyl acetate extracts derived from *A. bisporus, H. erinaceus, H. tessulatus, P. adiposa* and *P. eryngii* demonstrated antibiotic modulation when combined with ampicillin, amoxicillin and penicillin against a HA-MRSA isolate (Table 2). In contrast, no antibiotic modulation was observed with oxacillin, indicating that no tested extract was capable of counteracting the predominant resistance mechanism associated with oxacillin resistance, namely the low binding affinity of PBP2a.

**Table 2:** The antibiotic modulating properties of edible mushroom species ethyl acetate extracts against a HA-MRSA isolate

	MIC/(mg /mL)				
Species	Extract (¼ of antimicrobial MIC)	Antibiotic	Combination (Extract + antibiotic)	Fold-reduction	FICI score
	Ampicillin				
*Agaricus bisporus*	1.0	0.25	0.01	25.0	0.29^S^
*Grifola frondosa*	1.0	0.25	0.25	0	>1.0^I^
*Hericium erinaceus*	1.0	0.25	0.02	12.5	0.33^S^
*Hypsizygus tessulatus*	1.0	0.25	0.03	8.3	0.37^S^
*Lentinula edodes*	1.0	0.25	0.25	0	>1.0^I^
*Pholiota adiposa*	1.0	0.25	0.01	25.0	0.29^S^
*Pleurotus citrinopileatus*	0.25	0.25	0.25	0	>1.0^I^
*Pleurotus eryngii*	1.0	0.25	0.07	3.6	0.53^A^
*Pholiota microspora*	0.25	0.25	0.25	0	>1.0^I^
*Pleurotus ostreatus*	1.0	0.25	0.25	0	>1.0^I^
	Amoxicillin				
*Agaricus bisporus*	1.0	0.25	0.01	25.0	0.29^S^
*Grifola frondosa*	1.0	0.25	0.25	0	>1.0^I^
*Hericium erinaceus*	1.0	0.25	0.03	8.3	0.37^S^
*Hypsizygus tessulatus*	1.0	0.25	0.04	6.3	0.41^S^
*Lentinula edodes*	1.0	0.25	0.25	0	>1.0^I^
*Pholiota adiposa*	1.0	0.25	0.02	12.5	0.33^S^
*Pleurotus citrinopileatus*	0.25	0.25	0.25	0	>1.0^I^
*Pleurotus eryngii*	1.0	0.25	0.06	4.2	0.49^S^
*Pholiota microspora*	0.25	0.25	0.25	0	>1.0^I^
*Pleurotus ostreatus*	1.0	0.25	0.25	0	>1.0^I^
	Oxacillin				
*Agaricus bisporus*	1.0	0.0625	0.0625	0	>1.0^I^
*Grifola frondosa*	1.0	0.0625	0.0625	0	>1.0^I^
*Hericium erinaceus*	1.0	0.0625	0.0625	0	>1.0^I^
*Hypsizygus tessulatus*	1.0	0.0625	0.0625	0	>1.0^I^
*Lentinula edodes*	1.0	0.0625	0.0625	0	>1.0^I^
*Pholiota adiposa*	1.0	0.0625	0.0625	0	>1.0^I^
*Pleurotus citrinopileatus*	0.25	0.0625	0.0625	0	>1.0^I^
*Pleurotus eryngii*	1.0	0.0625	0.0625	0	>1.0^I^
*Pholiota microspora*	0.25	0.0625	0.0625	0	>1.0^I^
*Pleurotus ostreatus*	1.0	0.0625	0.0625	0	>1.0^I^
	Penicillin				
*Agaricus bisporus*	1.0	0.25	0.01	25.0	0.29^S^
*Grifola frondosa*	1.0	0.25	0.25	0	>1.0^I^
*Hericium erinaceus*	1.0	0.25	0.025	10.0	0.35^S^
*Hypsizygus* *tessulatus*	1.0	0.25	0.06	4.2	0.49^S^
*Lentinula edodes*	1.0	0.25	0.25	0	>1.0^I^
*Pholiota adiposa*	1.0	0.25	0.025	10.0	0.35^S^
*Pleurotus citrinopileatus*	0.25	0.25	0.25	0	>1.0^I^
*Pleurotus eryngii*	1.0	0.25	0.07	3.6	0.53^A^
*Pholiota* *microspora*	0.25	0.25	0.25	0	>1.0^I^
*Pleurotus ostreatus*	1.0	0.25	0.25	0	>1.0^I^

Modulating activity is classified into synergism (<0.5 (S)), additive (0.5-1 (A)), indifference (>1-4 (I)), and antagonism (>4 (ANT)), with reference to the FICI; *N*=3. MIC=minimum inhibitory concentration, FICI=fractional inhibitory concentration index

The observed FICI range (0.29–0.53) demonstrated by active extracts in conjunction with the ampicillin, amoxicillin and penicillin antibiotics indicates both synergistic and additive properties of tested edible mushroom extracts, while the remaining extracts demonstrated no modulating activity, resulting in indifference with respect to the FICI range (>1–4). *A. bisporus* exhibited the most consistent and strongest antibiotic modulatory activity, causing a 25-fold reduction in ampicillin, amoxicillin and penicillin MIC.

### Hericium erinaceus

In this study, the ethyl acetate extract exhibited the strongest overall antimicrobial activity against *S. aureus* (MIC=0.8 mg/mL). The antimicrobial activity of an *H. erinaceus* ethanol extract against *S. aureus* has been previously reported (MIC=2.5 mg/mL) (*19*), with the authors attributing the activity to the total phenolic content present. In the present study, the *H. erinaceus* ethyl acetate extract demonstrated an antimicrobial effect against MRSA (MIC=4 mg/mL) and a total phenolic value, expressed as GAE on dry mass extract basis, of 0.03112 mg/mg, potentially suggesting that other compounds may be responsible for the outlined activity.

Despite the suggested use of *H. erinaceus* as an antibiotic modulator (*20*), its modulating potential remains unknown. In this study, *H. erinaceus* demonstrated synergism (FICI≤0.5) with amoxicillin, ampicillin and penicillin against an MRSA isolate, with a maximum 12.5-fold MIC reduction (FICI=0.33) in combination with ampicillin. These results indicate that *H. erinaceus* may serve as a novel source of antibiotic-modulating compounds.

### Hypsizygus tessulatus

In the present study, the ethyl acetate extract of *Hypsizygus tessulatus* demonstrated antimicrobial activity against both *S. aureus* and MRSA (MIC=4 mg/mL). Previous studies have reported weak antimicrobial activity (44 % inhibition) of water extracts derived from *H. tessulatus* stalks (1.2 mg/mL) (*21*).

In addition to the outlined antimicrobial activity, *H. tessulatus* demonstrated synergism (FICI=<0.5) with ampicillin (FICI=0.37), amoxicillin (FICI=0.41) and penicillin (FICI=0.49), resulting in 8.3-, 6.3- and 4.2-fold MIC reductions, respectively. To our knowledge, this is the first study to report on the antibiotic-modulating effects of *H. tessulatus*, highlighting it as a potential novel source of antibiotic-modulating compounds.

### *Pleurotus* spp.

The ethyl acetate extracts of *Pleurotus citrinopileatus* were active against MRSA and *S. aureus* (MIC=1 mg/mL). Previous reports on *P. citrinopileatus* crude ethyl acetate extract indicate an antimicrobial effect against *S. aureus* (MIC=2.5 mg/mL) (*22*). The lipid metabolite glucosylceramide was suggested to be responsible for the outlined activity.

In the current study, despite showing no antimicrobial activity, *P. eryngii* ethyl acetate extract (1 mg/mL) reduced ampicillin MIC 3.6-fold (FICI=0.53) against MRSA, indicating additive properties (FICI=0.5–1). A previous study (*23*) reported synergism (FICI=<0.5) between a *P. eryngii* water extract (400 mg/mL) and ampicillin against a *S. aureus* isolate (FICI=0.19).

In this study, *P. ostreatus* extracts exhibited no antimicrobial or antibiotic-modulating properties. A previous study reported the antimicrobial activity of *P. ostreatus* mycelial extract (*V*(methanol):*V*(glycerol):*V*(water)=1:1:1) against *S. aureus* by zone-of-inhibition (disc diffusion) of (29.0±0.3) mm at an unreported concentration (*24*). However, it did not demonstrate antibiotic modulation with norfloxacin against *B. subtilis.* Aqueous ethanol (*φ*=60 %) extracts derived from *P. ostreatus* were evaluated for meropenem-modulating properties (25 mg/mL) against extended-spectrum β-lactamase-producing *E. coli* and *K. pneumoniae* isolates (*14*). A 4-fold reduction in meropenem MIC was reported; however, the extracts were not tested at subinhibitory concentrations, and synergism was defined using a FICI threshold of ≤1. In addition, the isolates exhibited no baseline resistance to meropenem prior to modulation. Similarly, a methanol (*φ*=80 %) extract derived from *Fistulina hepatica* (20 mg/mL) demonstrated synergistic activity (FICI=0.3) in combination with penicillin and ampicillin against MRSA (*25*). As in the previous study (*14*), subinhibitory concentrations of the mushroom extracts were not evaluated, reflecting differences in experimental design and criteria used to define synergism.

### *Pholiota* spp.

The antimicrobial and antibiotic modulating activity of species derived from the *Pholiota* family remain scarce. Thus, to the best of our knowledge, this is the first report on the antimicrobial (MIC=1 mg/mL) activity of *Pholiota microspora*.

In the present study, *P. adiposa* demonstrated inhibitory properties against both MRSA and *S. aureus* (MIC=4 mg/mL), while also exhibiting a 25-fold reduction in ampicillin MIC against MRSA (FICI=0.29), indicating synergism (FICI≤0.5). Previous studies evaluating the antimicrobial activity of ethanolic extracts derived from *P. adiposa* reported greater activity against MRSA (MIC=0.1 mg/mL) and *S. aureus* (MIC=0.2 mg/mL) (*26*). In that study, MIC values were determined using an agar dilution method, although the inoculum size was not specified. The relatively low abundance of phenolics in *P. adiposa* ethyl acetate extract, expressed as GAE on dry mass extract basis, ((0.0256±0.0002) mg/mg) in the present study suggests that non-phenolic compounds may be responsible for the reported activity.

### Agaricus bisporus

In this study, *A. bisporus* ethyl acetate extract demonstrated inhibition against both *S. aureus* and MRSA (MIC=4 mg/mL). *A. bisporus* methanolic (MIC=0.6 mg/mL) and ethanolic (MIC=0.03 mg/mL) extracts have previously demonstrated antimicrobial activity against *S. aureus* (*27*). Notably, the bacterial inoculum tested was 10^4^ CFU/mL, which is lower than that in this study (5·10^5^ CFU/mL) and might explain the variance. The *A. bisporus* ethyl acetate extract contained phenolics, expressed as GAE on dry mass extract basis, (0.0332±0.0004) mg/mg, which may indicate that phenolics are not responsible for the observed activity.

*A. bisporus* ethyl acetate extract was the most effective antibiotic-modulating extract, lowering the MIC of penicillin, ampicillin and amoxicillin to 0.01 mg/mL, a 25-fold reduction (FICI=0.29), indicating antibiotic synergism. A previous study demonstrated synergistic effects between *A. bisporus* ethanol and acetone extracts (25 mg/mL) and the anti-staphylococcal drug, AFN–1252 (0.0005 mg/mL), with the authors noting the potential application of *A. bisporus* as an antibiotic modulator (*28*). It was suggested that unsaturated fatty acids, in particular linoleic acid, were responsible for the outlined activity, most likely by disrupting the cell membrane and cell wall of the MRSA isolate. Synergism was inferred without the calculation of the FICI value, as the MIC of the *A. bisporus* extract was not determined.

**Fig. 1:**
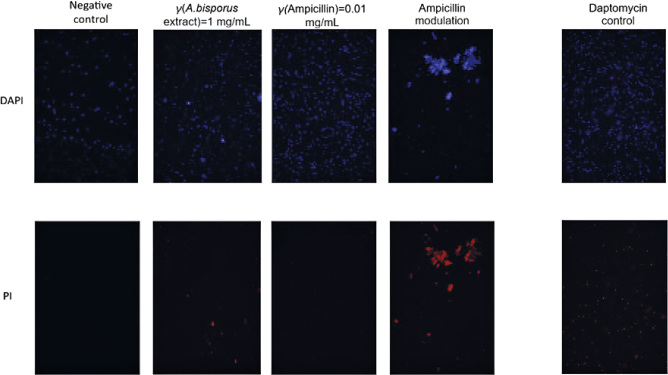
Cell viability assay of subinhibitory concentrations of *A. bisporus* ethyl acetate extract and ampicillin, and the subsequent antibiotic-modulating effects when combined 4′,6-diamidino-2-phenylindole (DAPI) region indicates live cells, while propidium iodide (PI) indicates membrane-compromised cells. Daptomycin was used as a positive control, while *φ*(dimethyl sulfoxide)=0.1 % was used as a negative control (*N*=3)

In this study, the ethyl acetate extract of *A. bisporus* has demonstrated increased cell membrane permeability through the cell viability assay (Fig. 1). When subtherapeutic concentrations of *A. bisporus* and ampicillin are tested individually, there is minimal effect on cell membrane damage. However, when both *A. bisporus* and ampicillin are used as a combination therapy, there is a notable increase in cell membrane leakage. Similarly, an ethanol extract derived from *A. bisporus* has previously shown cell membrane leakage against *S. aureus* (MIC=9.54 mg/mL) when used as an antimicrobial agent (*29*). This finding may illustrate the mechanism of action responsible for *A. bisporus* ability to modulate β-lactam antibiotics. It is suggestive that decreasing cell membrane integrity facilitates the localised concentration of antibiotic within the penicillin-binding protein domains, while simultaneously accelerating osmotic imbalance and subsequent cell lysis. However, no modulation was observed with oxacillin. Oxacillin resistance in MRSA is mediated by low β-lactam affinity for PBP2a while being resistant to the effects of β-lactamase, resulting in maximal activity to native PBPs. Consequently, an increase in membrane permeability or a reduction in β-lactamase activity would not be sufficient to enhance oxacillin activity, because the predominant resistance mechanism, PBP2a, remains unaffected.

*A. bisporus* is one of the most cultivated edible mushrooms globally, while it is estimated that 5–20 % of *A. bisporus* production generated is wasted due to physical abnormalities (*30*). While the repurposing of *A. bisporus* waste may serve as a sustainable source of effective antibiotic modulators, the approach has certain limitations for use in the food and biotechnology sectors. The active extract is obtained in low yields (0.48 % from dry mass; Table 3), which may cause difficulty for further purification and bioactivity-guided fractionation testing. In addition, the above results are obtained from a single MRSA isolate, which may not reflect the genetic and phenotypic diversity of MRSA strains, thereby limiting the broader applicability of the results. These factors indicate that further optimisation and testing against additional clinical isolates are needed before *A. bisporus* waste-derived antibiotic modulators can be translated into industrial or clinical applications.

**Table 3:** Extracted yields for each respective edible mushroom species, expressed as a percentage from starting dry mass (20 g)

	*Y*(extract)/%				
Species	Hexane	Methanol	Water	Ethyl acetate	Aqueous methanol
*Agaricus bisporus*	0.9	18.6	62.0	0.48	15.1
*Grifola frondosa*	1.3	19.9	71.3	0.31	14.4
*Hericium erinaceus*	1.8	32.2	54.0	0.45	20.2
*Hypsizygus tessulatus*	2.8	17.5	49.6	1.09	12.7
*Lentinula edodes*	0.9	23.7	62.1	0.5	20
*Pholiota adiposa*	1.8	25.2	61.5	0.37	17.6
*Pleurotus citrinopileatus*	1.2	22.9	65.9	0.56	17.4
*Pleurotus eryngii*	1.1	23.9	59.8	0.77	16.3
*Pholiota microspora*	0.9	31.7	55.5	0.26	24.5
*Pleurotus ostreatus*	1.5	22.3	67.3	0.35	17.6

Ethyl acetate and aqueous methanol extracts were derived from partitioning the methanol extract

## CONCLUSIONS

Of the minimal studies reporting on the antibiotic-modulating properties of edible mushroom species, there is considerable variation in the methods used for evaluating and classifying synergism, which further limits result comparison. This study has successfully outlined the antibiotic-modulating potential of ten edible mushroom species when combined with β-lactam antibiotics against a single MRSA isolate, with reference to recommended fractional inhibitory concentration index (FICI) classifications. Furthermore, this is the first study to assess the antibiotic modulating properties of *Hericium erinaceus, Hypsizygus tessulatus and Pholiota adiposa.*

The results presented in this study demonstrate the potential application of extracts derived from edible mushrooms as a source of antibiotic-modulating compounds. In particular, the antibiotic-modulating effects of *A. bisporus* has been demonstrated. It is hypothesised that *A. bisporus* ethyl acetate extract interferes with cell membrane integrity, leading to increased β-lactam uptake. The results of *Agaricus bisporus* ethyl acetate extract offer a potential use for β-lactam modulation against MRSA. Further research into the identification of the responsible analyte(s) is warranted.
